# Toxicogenomics Data: The Road to Acceptance

**DOI:** 10.1289/ehp.112-a678

**Published:** 2004-08

**Authors:** Kris Freeman

Questions abound regarding the use by regulatory agencies of data from microarray experiments. How does a regulator deal with risk assessment data that scientists are often unable to interpret—data that some companies are anxious to submit and others to withhold? How does this same regulator evaluate information that is produced without universally recognized standards for laboratory protocols or data formats? Pharmaceutical and chemical companies have their own questions. Do you submit all your data voluntarily, without knowing whether regulators will be able to understand it, and if and exactly how they will use it? Will submission of such complex data slow down approvals? What if data that cannot be interpreted now are later show to indicate toxicity, perhaps at levels that couldn’t be detected in animal testing? Could lawsuits follow? Regulatory penalties?

These and other questions have been discussed at numerous meetings between industry, environmental groups, the Food and Drug Administration (FDA), which regulates medications, and the Environmental Protection Agency (EPA), which regulates pesticides significantly in the last two years, according to John Leighton, supervisory pharmacologist in the Division of Oncologic Drug Products of the FDA Center for Drug Evaluation and Research (CDER). At an FDA-sponsored workshop held in May 2002, Leighton says, the take-home question was whether microarray technology was sufficiently developed for scientific purposes. Within 18 months, at a follow-up workshop in November 2003, he says, “the issues had shifted from ‘Is the technology useful?’ to *How* is it useful?”

Proving that microarray data, or “expression signatures,” can be valid measures of environmental exposure is a major accomplishment of a 1999–2003 research program by the Health and environmental Sciences Institute. The HESI experiments, published in the March 2004 toxicogenomics issue of *EHP*, showed the patterns of gene expression detected in microarray experiments can give insight into biologic mechanisms, and can even distinguish between damage found in different cell types. Although most expression signatures still can’t be interpreted or linked to biologic effects, officials at both the FDA and the EPA express optimism that the use of microarray data could help them better protect public health. Industry however, taken as a whole, may not be quite so sure.

Efforts to encourage communication between regulators and industry have included workshops held by the National Research Council (NRC) Committee on Emerging Issues and Data on Environmental Contaminants, which is funded by the NIEHS. Since its establishment in April 2002, the committee has held seven workshops to discuss issues related to the future use of toxicogenomics data in government risk assessment and regulatory decision and policy making. These issues include the many challenges that remain to be resolved before these tools find direct application in chemical risk assessment, says David Eaton, chair of the NRC committee and director of the NIEHS Center for Ecogenetics and Environmental Health at the University of Washington.

Because of the expense involved in running microarray experiments, including the costs of analyzing data, microarrays are generally not used in detailed dose and time-course studies, says Eaton. As a result, current microarray data often provide a limited snapshot of information that Eaton says can be very useful in terms of generating hypotheses about mechanisms of exposure, although the application of such information for regulatory purposes is fraught with uncertainty.

We think there are powerful uses for genetic data, including microarray data, in the real world of drug safety, to both test products and do “forensic” studies—that is, go back and investigate safety problems after marketing. –Janet Woodcock, CDER

## Government’s Take on Microarray Data

“We think there are powerful uses for genetic data, including microarray data, in the real world of drug safety, to both test products and do ‘forensic’ studies—that is, go back and investigate safety problems after marketing,” says CDER director Janet Woodcock. “In cases of some adverse drug effects, companies may be able to go and look for specific genotypes that are distinctive and at risk for an adverse event.” Agency regulators, she adds, hope that intractable drug toxicity problems, such as hepatotoxicity, could be solved through microarray or gene expression technologies.

EPA representatives hope that microarray technology, along with proteomics and metabolomics experiments, will help the agency better screen the vast number of chemicals it is mandated to regulate. Using traditional tests, it can easily take 3–4 years and $20 million to test the toxicity of a pesticide, says Robert Kavlock, director of the Reproductive Toxicology Division in the EPA Office of Research and Development. “In the long run, we expect that the use of ‘omics’ technologies can be applied to a variety of bioassays, some *in vitro*, some *in vivo*, that will help us prioritize chemicals for testing in the more lengthy, expensive, and animal-intensive testing batteries, and perhaps even to guide selection of which tests should be done within those batteries,” says Kavlock. “By doing so, we will become more efficient and effective in our utilization of animal tests.”

“Genomics won’t replace animal testing, not yet,” adds William Benson, director of the Gulf Ecology Division of the EPA National Health and Environmental Effects Research Laboratory. “But we hope it will allow us to use animals more wisely.”

Microarrays and other “omics” technologies could also be used in environmental monitoring, such as water testing. As the technology decreases in cost, local regulators may be able take a microarray chip into the field, apply water samples, and get an answer right there regarding the presence of bacteria, viruses, and other pathogens, according to Kerry Dearfield, senior scientist for science policy in the EPA Office of the Science Advisor. Dearfield, along with Benson and Kathryn Gallagher, science policy council staff in the Office of the Science Advisor, wrote a March 2004 EPA draft white paper on the impact of genomics technologies on EPA regulatory activities.

Although both the EPA and the FDA have discussed possible uses of microarray data, only the FDA has issued requirements for the submission of such data. At press time the agency was working on a final version of its “Guidance for Industry: Pharmacogenomic Data Submission,” released in draft form in November 2003. Under the draft guidance, companies may be required to submit microarray data used to determine differential dosing of a medication by genotype during development (a requirement that applies to animal testing as well as human clinical trials). The guidance also encourages, but does not require, companies to develop suitable genetic tests for such medications to allow physicians to determine if a drug is appropriate for a given patient.

“The centers for drugs and devices are working together for the development of drug–device combinations,” says Atiqur Rahman, acting deputy director for the CDER Division of Pharmaceutical Evaluation 1. “If a drug’s approval becomes based upon a specific test, you can’t approve the drug unless the test is available.”

The draft guidance also encourages, but again does not require, voluntary submission of microarray data from exploratory studies such as experiments to screen multiple compounds for possible toxicity or efficacy. Companies are also asked to supply research data resulting from general gene expression analyses in cells, animals, and humans, as well as analysis of single-nucleotide polymorphisms in trial participants.

In addition, all data on “known valid biomarkers,” including those collected during exploratory studies, must be submitted to the FDA. Although the guidance does not specify the types of biomarkers that must be submitted, Woodcock clarifies that the agency is mainly interested in so-called safety biomarkers, those that indicate toxicity. “Companies don’t have to submit any data on nonclinical efficacy biomarkers,” she says.

Currently, the EPA’s official dictum on the regulatory use of microarray data is limited to a four-page “Interim Policy on Genomics” issued in June 2002. The interim policy states that microarray data are expected to be valuable, and that they “may be received as supporting information for various assessment and regulatory purposes, e.g., identifying an environmental stressor’s mode or mechanism of action.” But the interim policy does not provide any details on potential required submissions of gene expression data. There is no current effort at the EPA to expand or update the interim policy.

## What It Means for Industry

Industry response to these regulatory efforts ranges from enthusiasm to extreme caution. “Some companies will not test a drug with a microarray experiment that has any chance of becoming part of a regulatory package,” says Kurt Jarnagin, vice president for biological sciences and chemical genomics at Iconix Pharmaceuticals. “And then there are companies who view [submission of microarray data] as a positive, who say the FDA gets more information, we get more information, and we might find a positive aspect to our drug that we didn’t know about.”

There are already a number of drugs approved for people with specific genetic variations. Most are powerful cancer drugs for which the boundaries between efficacy and toxicity are narrow. One example is imatinib mesylate (trade name Gleevec), which is approved for patients with a specific type of leukemia characterized by a chromosomal rearrangement in the cancerous cells. Another example is trastuzumab (trade name Herceptin), an intravenous treatment for advanced metastatic breast cancer. Trastuzumab is effective in treating tumors that produce excess amounts of the HER2 protein, a tyrosine kinase receptor.

In addition to determining who is most likely to respond to a drug, genetic studies could also be used to screen out those most susceptible to toxic side effects. One example is the case of the lung cancer drug gef-tinib (trade name Iressa), which inhibits a tyrosine kinase that is overexpressed in non–small cell lung cancer, the leading cause of cancer deaths in the United States. After the drug was approved, the FDA received reports of severe, sometimes fatal, toxicity in 0.3–2.0% of patients receiving the drug. In addition, during clinical trials, the drug was effective in only 10–19% of persons with non–small cell lung cancer. Preliminary results published 20 May 2004 in the *New England Journal of Medicine* indicate that the drug is effective only in people who have heterozygous mutations in the tyrosine kinase epidermal growth factor receptor, coded by the gene *EGFR*.

Microarray data were not submitted during the approval process for any of these drugs, but could be used in the future to help develop population-specific treatments, according to Rahman. “A certain type of gene expression constituting a gene signature may help determine if a person is a candidate for treatment with a particular drug and is likely to respond to the therapy,” he says.

In contrast to the pharmaceutical industry, “the chemical industry is not chomping at the bit to use toxicogenomics data,” claims Linda Greer, director of the Natural Resources Defense Council public health program and a member of the NRC committee. “The status quo works better for them rather than a system where chemicals can be screened systematically,” she adds. More than 90% of the industrial chemicals in commerce have not been tested for their toxicity, Greer says, and better screening might cause increased scrutiny of such compounds under the Toxic Substances Control Act (TSCA) and the Federal Insecticide, Fungicide, and Rodenticide Act (FIFRA).

In the long run, we expect that the use of “omics” technologies can be applied to a variety of bioassays, some in vitro, some in vivo, that will help us prioritize chemicals for testing in the more lengthy, expensive, and animal-intensive testing batteries, and perhaps even to guide selection of which tests should be done within those batteries. –Robert Kavlock, EPA

TSCA gives the EPA authority to require reporting or testing of industrial chemicals that may pose an environmental or human health hazard, and to ban the manufacture and import of chemicals that pose high risks. However, the EPA is not required—nor does it have the resources—to perform extensive toxicity testing on every industrial chemical available for sale in the United States. Nor can chemical companies increase their profits by determining genetically based differences in responses to their general-use products. “What we sell is going to be out there for the general population to use, so we’re compelled to protect the most sensitive individual,” says George Daston, a senior toxicologist in the Central Product Safety group of Procter and Gamble.

Risk exposure testing for industrial chemicals can also be less straightforward than pharmaceutical testing, increasing challenges for both industry and the EPA. In contrast to pharmaceuticals, which people generally are exposed to at known doses for intended biologic effects, environmental exposures to industrial chemicals among the general public are often quite low. In addition, people are often exposed to mixtures of compounds—for example, to several pesticides from a piece of fruit, or to hundreds of chemicals from swimming near a storm sewer outfall. As a result, singling out the effects of a single industrial compound can be extremely difficult.

Nevertheless, some chemical companies are conducting microarray experiments to better understand mechanisms of toxicity, which could lead to better risk assessment information regarding susceptible populations, co-mixtures of chemicals, and low levels of exposure, according to Greer and Dearfield. For example, Greer says, microarray studies could build on research published in the August 2004 issue of *EHP* linking exposure to the complex mixtures of disinfection by-products in drinking water and low birth weight in children of women with polymorphisms in the *CYP2E1* and *C677T* genes.

Research such as this raises tough regulatory issues for the EPA, Greer adds—is the EPA going to lower the standard of disinfection by-products to protect what might turn out to be a substantial group, or are they going to warn people and tell them to get tested for genetic susceptibility? “Our answer,” she says, “is that regulators need to protect the most susceptible individuals. You can’t tell people not to drink water or to buy bottled water.”

Microarray data may also be able to detect cellular activity in whole animals at levels far lower than those that cause discernible changes such as tumors or weight loss. In recent studies, researchers at the Microarray Center of the NIEHS National Center for Toxicogenomics (NCT) detected early indicators of mitochondrial damage before any adverse effect could be detected through traditional toxicity tests, says Microarray Center director Richard Paules. Such research doesn’t necessarily indicate that such low doses are toxic, says Paules, “but these gene expression changes could be an indication that higher or longer exposures have the potential to cause adverse effects and should be studied more closely.”

Some companies will not test a drug with a microarray experiment that has any chance of becoming part of a regulatory package. And then there are companies who view [submission of microarray data] as a positive, who say the FDA gets more information, we get more information, and we might find a positive aspect to our drug that we didn’t know about. –Kurt Jarnagin, Iconix Pharmaceuticals

Signatures of mechanisms of toxicity in different species may also improve researchers’ ability to compare the results of animal studies and human health outcomes. A chemical that causes, say, cancer in a rat may not have the same effect in people if the two species process the compound differently. Such research is especially important for the manufacturers of industrial chemicals, who do not test their products on humans, according to Jim Bus, director of external technology at The Dow Chemical Company and a member of the NRC committee.

## Concerns Voiced

Although the use of microarray data and acceptance by regulators can be beneficial for pharmaceutical and other chemical manufacturers, many industry representatives still express concerns about the use of such data by the FDA and the EPA. One of these concerns—which runs contrary to the optimism expressed by government—is that submission of complex expression data will slow processing and approval of applications rather than streamline the process. Current FDA approval times for regular drug applications are about 18 months, down from 30 months several years ago. Approval for priority drugs with a public health benefit, such as AIDS medications, can take as little as 6 months, according to Woodcock.

Pharmaceutical and chemical companies have also expressed concern that the FDA and the EPA might overreact to microarray data. The primary issue, according to Daston, is determining what type of signature constitutes an adverse effect; the challenge is to distinguish adverse responses to a chemical exposure from homeostatic responses—that is, normal changes that may indicate a cell is disposing of a toxicant in a way that will not lead to lasting damage or that may not be related to the exposure at all.

Dearfield explains further: “Gene expression changes all the time. You can walk from a dark room into the sunlight, and you’re going to get all kinds of genomic signature changes. Is that bad? No—it’s the way the body normally operates. You need to sort out that kind of change from a change caused by an adverse stressor.”

Similarly, there is concern that the agencies will be “excessively reactive” to single gene changes, says Jarnagin. “Hypothetically you do a microarray expression on a potential drug’s effect, and lo and behold, the oncogene RAS is elevated five- or tenfold. Using a [toxicogenomics] database, you can see that there are many approved drugs that elevate RAS. Every drug in our database elevates at least one known oncogene. None of these drugs are known to cause cancer at therapeutic doses.”

Although the FDA draft guidance states that voluntary submissions of data will not be used for regulatory purposes, some companies still are reluctant to part with the results of exploratory microarray experiments. Some companies fear that proprietary data from one application will be used to judge data in another. In response, Woodcock says, “We cannot apply proprietary data to another application; we can’t make it public.” However, she says, regulators do learn from the reviews they conduct. And although they can’t directly compare data from one application to another, problems they see in one application might cause them to more carefully scrutinize another application with similar results.

Legal concerns include the potential for being sued if microarray data that couldn’t be interpreted at the time of submission later turn out to indicate toxicity in some people or under some conditions. The fear of lawsuits is such that some companies haven’t gone to the next stage in using microarrays for evaluating the effects of drugs under development, for either good or bad effects, says Roger Ulrich, president of Rosetta Inpharmatics, a subsidiary of Merck and Company.

Manufacturers of industrial chemicals are also concerned about EPA penalties. If a company discovers a previously unknown adverse effect for a given chemical, the company is required under TSCA to submit a report to the EPA within a few days, says Bus. The same is true if toxicity is detected at concentrations lower than previously found. “Say you’re dosing animals with a chemical where, historically, an effect has not been seen below a dose of ten milligrams per kilogram,” Bus explains. “You do another study and suddenly, you find a unique effect at one milligram per kilogram. Under TSCA, you’re required to report that.” If such a report were delayed because the significance of the microarray data wasn’t understood at the time of testing, manufacturers could conceivably face retroactive fines and penalties. If penalties are levied per day and a significant amount of time has passed, fines can be substantial, says Bus. Under TSCA, the EPA has the authority to levy fines of up to $27,500 per day for nondisclosure of required information.

## Fine-tuning the Process

The FDA is working to alleviate some of industry’s concerns. To facilitate its ability to handle microarray data and keep approvals moving, the agency has collaborated with private firms on training exercises. Through a material transfer agreement, Iconix has given the CDER access to its proprietary relational toxicogenomics database, DrugMatrix, for evaluative and educational purposes, says Karol Thompson, molecular toxicology team leader in the CDER Division of Applied Pharmacology Research. The DrugMatrix database contains expression information related to more than 600 substances, including many approved medications. Iconix also led two workshops on microarray technology in February 2003 and January 2004 for members of the Nonclinical Pharmacogenomics Subcommittee of the CDER Pharmacology/Toxicology Coordinating Committee. The pharmacogenomics firm Gene Logic also has provided the FDA with expression data from its proprietary GeneExpress system database as part of a collaborative project with CDER research scientists and statisticians to identify endogenous genes that can serve as indicators of microarray sample quality.

In addition, the FDA worked with the company Expression Analysis on a mock submission using toxicology data developed by Schering-Plough Corporation for a candidate drug that did not go on to clinical trials. The submission included microarray data, histology data, clinical chemistry data, and phenotype data. The exercise served as a practice run to help the FDA understand the format and content of future drug submissions containing microarray data.

“I think the FDA, Expression Analysis, and Schering-Plough gained a tremendous amount from this collaboration,” says Steve McPhail, CEO of Expression Analysis, which provides commercial microarray testing, analysis, and data management services. “We gained a great perspective in working with the FDA and in beginning to understand their thinking on how this type of data should be formatted for future regulatory submissions. And I think the FDA gained value from the submission from our experience with lots of clients and users of data and the way that they need to become prepared for submission.”

Although regulators and industry are working hard to hammer out the issues around the submission of gene expression data, such submissions are still somewhat premature, says William Mattes, a researcher on the HESI effort and senior scientific director of toxicogenomics at Gene Logic. For example, researchers and regulators have not yet even decided how to report data. The ultimate goal is to “submit data in some tabular format that is computer-friendly and will allow regulators to crunch the data, analyze it with software,” he says. “We have not seen the FDA truly, openly discuss what data standards would be. . . . The issue is hugely in flux.”

Mattes serves on a committee on pharmacogenomics standards sponsored by the Interoperable Informatics Infrastructure Consortium, Health Level Seven, and the Clinical Data Interchange Standards Consortium, nonprofit organizations developing data standards for health care and clinical trials. According to Mattes, the joint committee is discussing high-level questions regarding the kind of data that should be included in microarray submissions. Other groups are promoting the use of specific data formats such as the MIAME (Minimum Information About a Microarray Experiment) standards for content, as well as the accompanying MAGE (MicroArray and Gene Expression) data format standards developed by the Microarray Gene Expression Data Society. The European Bioinformatics Institute, the NCT, and HESI have proposed definitions for MIAME/Tox, which would add toxicogenomics annotations to the basic MIAME content framework.

The chemical industry is not chomping at the bit to use toxicogenomics data. The status quo works better for them rather than a system where chemicals can be screened systematically. –Linda Greer, Natural Resources Defense Council

Agreement on data formats will do industry and regulators little good if experimental protocols are weak or inconsistent. The HESI studies found significant variation among results of microarray experiments that were caused by differences in procedures among participating laboratories, including different operating procedures for isolating and labeling mRNA samples, nonstandard settings on hardware and software, and differences in gene coverage and annotation across different technology platforms. Jarnagin and others say the standardization problems found in the HESI experiments, some of which were conducted 3–5 years ago, are not as serious now. “There’s been substantial advancement in the field in the last few years,” says Jarnagin.

The quality and consistency of microarray chips has improved since the HESI experiments were conducted, agrees Brenda Weis, who along with William Suk administers the Toxicogenomics Research Consortium (TRC), a component of the NCT. “The commercial products are particularly good,” she says. “The manufacturing is at a very high level.”

Testing different microarray types was an important part of initial standardization experiments by the TRC, which involves researchers at five academic centers across the country, as well as the NIEHS Microarray Center. The consortium’s work builds on the HESI studies by systematically addressing different steps of the microarray experiment to see where variability is most likely to be introduced, says Weis.

In the consortium’s first set of experiments, reported in the March 2004 toxicogenomics issue of *EHP*, the centers used a total of 12 different microarray platforms. In the multifaceted experiments, all six consortium centers used two common platforms: an oligo microarray manufactured at one of the centers and the commercial Agilent mouse microarray platform, developed by TRC investigators working collaboratively with Agilent and the NCT microarray resource contractor, Paradigm Genetics. There were also 10 other “resident” cDNA- or oligo-based platforms that were manufactured at and used by the individual centers.

Other variables addressed in the experiments have included the use of spike-in RNA and RNA reference samples, known sequences of RNA used as controls in microarray experiments (spike-in RNA is added to samples at a known concentration whereas reference RNA is kept separate from the samples but run through the same microarray experiment). The goal was “to see if they provided utility in helping us understand how the different platforms performed,” says Paules.

There are still other aspects of microarray analysis that can introduce variability into results, according to Weis. During the TRC studies, as during the HESI studies, researchers found that the way each individual center handled the RNA—including the labeling of the samples, the hybridization and wash conditions, and variables in the scanning and analysis—all had an impact on the eventual outcomes, says Paules. Results and recommendations for improving standardization have been submitted for publication.

Now that studies have addressed the technology, the consortium has begun another series of experiments focusing on the replication of genomic signatures. Each center will receive common reference RNA samples, Agilent microarray chips, and compounds (acetaminophen and its nontoxic isomer) to test using experimental animals. All of the centers will use standardized protocols for the microarray analyses. The hope, says Weis, is “to standardize the technical aspects of the experiment in order to address the issue of reproducibility of the biological response across multiple research groups. Whether or not we can do this successfully is important information for the regulatory community.”

Other groups that are studying method standardization include the External RNA Controls Consortium, a volunteer group sponsored by the National Institute of Standards and Technology. The group is working to develop methods to evaluate the performance of gene expression assays based on the measurement of external RNA controls, such as spike-in controls.

Standardization of animal models is another concern in microarray experiments. Researchers with the National Toxicology Program (NTP), an interagency organization based at the NIEHS, are studying changes in microarray results caused by homeostatic responses in Fisher 344 rats, one of the primary animal models used by the NTP. Results thus far, currently in press at *Toxicologic Pathology*, show differences in microarray signatures in samples taken from the left lobe of the liver compared to those from the median liver lobe of the same animal.

“You may get the same overall story from the two samples, but not the same number of genes or the same intensity of expression,” says Gary Boorman, a research scientist with the NTP and the NIEHS Environmental Toxicology Program, and a coauthor of the forthcoming paper. These results indicate that when labs coordinate their efforts, they should not only look at the technical issues, such as the microarray platforms each group is using, but also make sure that their methods for sampling animal models are uniform, says Boorman.

The NTP group is also studying variables including the time of day that tissue is collected, and the life stage and sex of the animal. The goal is to describe how normal variability in an animal strain can affect the interpretation of studies using microarray technology, says Nigel Walker, chair of the NTP’s toxicogenomics faculty and a staff scientist with the NIEHS Environmental Toxicology Program. “We’re trying to define ‘normal,’” says Walker, “so we know when the change in a gene is beyond the range of normal physiological variability.”

## The Burden of Interpretation

Once results of microarray experiments are reproduced, scientists and regulators are still faced with the difficulty of interpreting genomic signatures. “There seems to be a lack of consensus on how data should be analyzed,” says Timothy Zacharewski, an assistant professor of biochemistry and molecular biology at Michigan State University and a member of the NRC committee.

For example, although the FDA draft guidance requires the submission of all data on “known valid biomarkers,” the agency currently does not recognize any genomic signatures as valid biomarkers, according to Leighton. “A lot of stuff has been published, but not all of it is of the same quality, even though it’s in the peer-reviewed literature, either because of a small population study or inadequate controls,” he says.

The FDA draft guidance does not specify how genomic signatures are to be validated as biomarkers. “It’s an important issue, and we’re discussing that,” says Leighton. “In the near future, we may need to come out with guidance on how to validate a genomic signature.” However, he says he doesn’t anticipate such guidance being issued soon; the agency doesn’t want to act in haste lest a less-than-optimal procedure be institutionalized prematurely.

There are logistical questions to consider. “How do you validate a safety biomarker, say for liver injury? You can’t run a clinical trial where you cause liver injury,” says Ulrich. Instead, scientists must compare genomic signatures to traditional toxicity tests using animals. But some traditional biomarkers can be subjective and often equivocal, says Ulrich. He cites the examples of alanine aminotransferase and aspartate aminotransferase, biomarkers of liver injury that also are occasionally associated with muscle injury. “Weight lifters and long-distance runners express [these enzymes],” he says. “They’re subjective biomarkers because they’re not liver-specific.” (Leighton notes, however, that the biomarkers industry and academia rely upon the most are less subjective. “Every lab uses the same core set,” he says, “and they’ve been in use for many years.”)

Private companies have little motivation to validate safety biomarkers, in part because they don’t know how they’ll be used, says one pharmaceutical representative who asked to remain anonymous. “It’s expensive to validate a genomic signature. And if we make the investment and develop a better biomarker for toxicity, all it will do is make it tougher to get approvals.” As a result, the bulk of validation efforts probably will be conducted and disseminated by nonprofit groups, academia, and government labs.

One leader in this area is the NCT. In addition to studies of experimental protocols and replicability, the NCT is also studying signatures generated by specific exposures. The NCT’s Microarray Center is focusing on liver toxicants such as acetaminophen [see “Phenotypic Anchoring: Linking Cause and Effect,” *EHP* 111: A338–A339 (2003)]. Other government facilities are contributing as well. EPA research into gene expression includes studies of sentinel species such as amphibians, fish, and aquatic microbes. The Department of Energy is using microarray experiments and other techniques to study microbial communities used in the remediation of toxic waste. In the nonprofit realm, the public ArrayExpress database of expression data, managed by the European Bioinformatics Institute, contains all the results from the HESI experiments as well as expression data from other studies.

There is also at least one commercial company that is contributing to public domain information on safety biomarkers. In March 2004, Iconix announced plans to publish five expression signatures of drug-induced toxicity in the liver, kidney, and heart. Information on one of the signatures, for injury to renal tubules, was presented that month at the annual meeting of the Society of Toxicology.

As industry and regulators wrestle with the intricacies of microarray data formats and submission, even more complex challenges loom: the data produced by proteomics and metabolomics research. “We’re well aware that metabolomic and proteomic data might be more important in the long term than the genomic data,” says Leighton. Among other reasons, samples are more readily available; it’s easier to collect blood and urine than to take a liver biopsy. “Then again,” says Zacharewski, “with proteomics and metabolomics you still have the problem of large, complex data sets of which only a fraction can be interpreted as being linked to any biological effect.”

Issues of standardization and validation will be similar for all of the “omics” technologies. So will tensions between concerns of industry and statutory obligations of regulators. That means many more meetings between industry and agencies. “We’re absolutely committed to not setting standards in isolation,” says Benson. “It is essential for the agencies to work together and with industry and academia when developing this regulatory framework.”

## Regulatory Resources

### GROUPS

**FDA Center for Drug Evaluation and Research (CDER)**

The CDER reviews applications for new prescription and over-the-counter drugs to ensure they been adequately tested and are safe for human use. The CDER also monitors drugs that are already on the market for unexpected health risks. **http://www.fda.gov/cder/**

**National Research Council (NRC) Committee on Emerging Issues and Data on Environmental Contaminants**

This committee provides a public forum for government, industry, environmental groups, and the academic community to discuss emerging evidence and issues in toxicogenomics, environmental toxicology, risk assessment, exposure assessment, and other related fields. **http://dels.nas.edu/emergingissues/index.asp**

**Microarray Gene Expression Data (MGED) Society**

This international group of biologists, computer scientists, and data analysts aims to facilitate microarray data sharing by establishing standards for data annotation and exchange, fostering the creation of microarray databases and related software implementing these standards, and promoting the sharing of high-quality, well-annotated data within the life sciences community. The group hopes to extend this mission to other “omics” technologies. **http://www.mged.org/**

### DOCUMENTS

**Guidance for Industry: Pharmacogenomic Data Submission**

This guidance, issued by the FDA in draft form in November 2003, contains nonbinding recommendations on the submission of pharmacogenomics data during the drug application process. **http://www.fda.gov/cder/guidance/index.htm** [select “Pharmacogenomic Data Submissions” under the heading “Procedural (Draft)”]

**Interim Policy on Genomics**

This four-page policy paper outlines the EPA’s standing position on the relevance and use of genomics technologies in risk assessment. **http://epa.gov/osa/spc/htm/genomics.pdf**

**Mini-Monograph: Genomics and Risk Assessment**

This mini-monograph published in the March 2004 toxicogenomics issue of *EHP* includes recommendations for conducting studies and handling data based on studies by the Health and Environmental Sciences Institute/International Life Sciences Institute. **http://ehp.niehs.nih.gov/txg/docs/2004/112-4/toc.html?section=toxicogenomics**

**Potential Implications of Genomics for Regulatory and Risk Assessment Applications at EPA**

This March 2004 draft white paper by the EPA examines the possible impact of genomics technologies on agency regulatory activities. **http://www.epa.gov/osa/genomics-external-review-draft.pdf**

### OTHER RESOURCES

**ArrayExpress**

This public database of expression data is managed by the European Bio-informatics Institute. **http://www.ebi.ac.uk/arrayexpress/**

**External RNA Controls Consortium Workshop: Specifications for Universal External RNA Spike-In Controls**

The External RNA Controls Consortium, sponsored by the National Institute of Standards and Technology, has posted presentations from this bioinformatics workshop on its website. The consortium is working to develop methods to evaluate the performance of gene expression assays based on the measurement of external RNA controls. **http://www.cstl.nist.gov/biotech/workshops/ERCC2003/**

**MAGE (MicroArray and Gene Expression)**

This page of the MGED Society website provides MAGE-related links, tools, and other resources. **http://www.mged.org/Workgroups/MAGE/mage.html**

**MIAME (Minimum Information About a Microarray Experiment)**

This page of the MGED Society website describes MIAME principles and requirements, and lists links to relevant tools and news. Also includes a document by the European Bioinformatics Institute, the NIEHS National Center for Toxicogenomics, and the Health and Environmental Sciences Institute/International Life Sciences Institute in which these groups propose definitions for MIAME/Tox, which would add toxicogenomics annotations to the basic MIAME content framework. **http://www.mged.org/Workgroups/MIAME/miame.html**

## Figures and Tables

**Figure:**
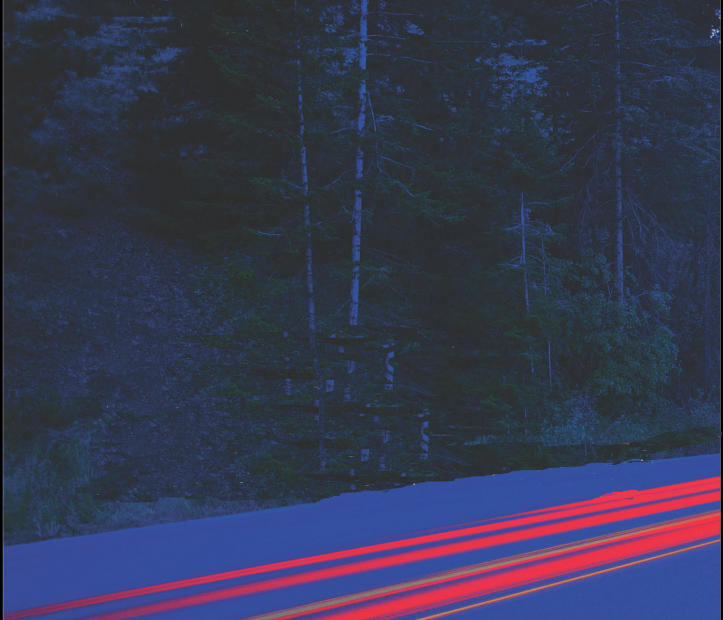


**Figure:**
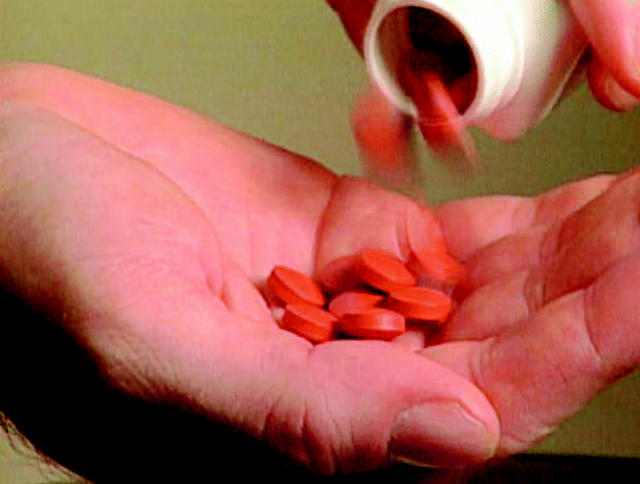
**Drug data dilemma.** Researchers and regulators alike are struggling with the complexities—and uncertainties—of toxicogenomics data.

**Figure:**
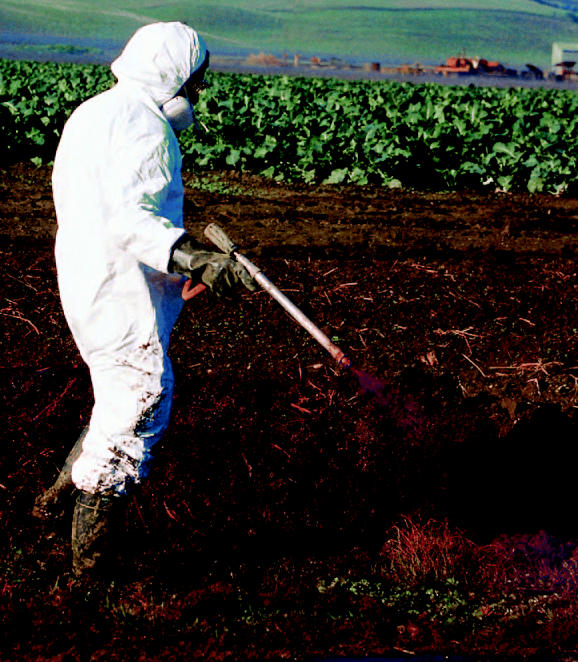
**Chemical conundrum.** The EPA is moving cautiously toward considering toxicogenomics data in chemical regulation.

